# Anticancer response to disulfiram may be enhanced by co-treatment with MEK inhibitor or oxaliplatin: modulation by tetrathiomolybdate, KRAS/BRAF mutations and c-MYC/p53 status

**DOI:** 10.3332/ecancer.2019.890

**Published:** 2019-01-08

**Authors:** Ali Calderon-Aparicio, Alejandro Cornejo, Andrea Orue, Manuel Rieber

**Affiliations:** Instituto Venezolano de Investigaciones Cientificas, Tumor Cell Biology Laboratory, Caracas 1020-A, Venezuela; * These authors contributed equally to this work.

**Keywords:** Cu chelation, Cu-MEK activation, KRAS/c-MYC, p53 status, oxaliplatin, tetrathiomolybdate, disulfiram

## Abstract

Ammonium tetrathiomolybdate (TTM) and disulfiram (DSF) are copper (Cu) chelators in cancer clinical trials partly because Cu chelation: a) restricts the activity of Cu-binding MEK1/2 enzymes which drive tumourigenesis by KRAS or BRAF oncogenic mutations and b) enhances uptake of oxaliplatin (OxPt), clinically used in advanced KRAS-mutant colorectal carcinomas (CRC). Whereas TTM decreases intracellular Cu trafficking, DSF can reach other Cu-dependent intracellular proteins. Since the use of individual or combined Cu chelation may help or interfere with anti-cancer therapy, this study investigated whether TTM modifies the response to DSF supplemented with: 1) UO126, a known MEK1/2 inhibitor; 2) other Cu chelators like neocuproine (NC) or 1, 10-o-phenanthroline (OPT) in wt p53 melanoma cells differing in BRAF or KRAS mutations; 3) OxPt in mutant p53 CRC cells devoid of KRAS and BRAF mutations or harbouring either KRAS or BRAF mutations. TTM was not toxic against ^V600E-^*^mut-BRAF^* A375 and ^G12D-mut-^*^KRAS/high c-myc^* C8161 melanoma cells. Moreover, TTM protected both melanoma types from toxicity induced by DSF, NC and co-treatment with sub-lethal levels of DSF and the MEK inhibitor, UO126. Toxicity by co-treatment with DSF+OPT was poorly reversed by TTM in C8161 melanoma cells. In contrast to the greater toxicity of 0.1 μM DSF against mutant p53 CRC cells irrespective of their KRAS mutation, TTM did not protect ^G12V-mut-^*^KRAS/high c-myc^* SW620 CRC from DSF+OxPt compared to ^KRAS-WT/BRAF-WT^ Caco-2 CRC. Our results show that DSF co-treatment with: a) MEK inhibitors may enhance tumour suppression; b) OxPt in CRC may counteract impaired response to cetuximab by KRAS/BRAF mutations and c) as a single treatment, TTM may be less effective than DSF and decreases the efficacy of the latter.

**Highlights:**

- Potentiation of melanoma antitumour toxicity of DSF by MEK inhibitor is reversed by TTM.
- KRAS/c-MYC dysregulation attenuates TTM reversion of melanoma toxicity by DSF + OPT.
- KRAS/c-MYC dysregulation increases melanoma NC toxicity reversed by TTM.
- BRAF mutation and lower c-MYC may attenuate toxicity by DSF ± OxPt in colorectal cancer cells

## Introduction

The copper (Cu) dependence of MEK1/2 dysfunctional signalling is an important target to inhibit tumour cells with BRAF or KRAS mutations [1, 2]. In BRAF-(V600E)-mutated melanoma, pharmacological Cu sequestration with a clinically used copper chelator, ammonium tetrathiomolybdate (TTM) inhibits MEK1/2 kinase activity and reduces mutant BRAF-driven growth in melanoma cell lines resistant to BRAF or MEK1/2 inhibitor [3]. In contrast to the initial response rates (>50%) of BRAF inhibitor monotherapy in *BRAF*^V600^-mutant melanoma, approximately 5% of patients with *BRAF*^V600E^ colorectal cancer respond. Preclinical studies suggest that the lack of efficacy in *BRAF*^V600E^ colorectal cancer is due to adaptive feedback reactivation of mitogen-activated protein kinase signalling, often mediated by epidermal growth factor receptor (EGFR) [4]. Diminished response to treatment with anti-EGFR monoclonal antibodies is found in KRAS-mutant or BRAF-mutant colorectal cancer [5–8] but these mutations do not impair response to oxaliplatin- (OxPt) or cisplatin-based chemotherapy [9, 10]. Response to platinum (Pt)-based anti-cancer drugs involves enhancing its uptake, through the copper (Cu) transporter protein hCTR1 [10, 11] whose expression is increased by Cu chelators like TTM. The latter has been touted as an anti-cancer agent [3], [12–15] because its Cu chelation enhances uptake of cisplatin or OxPt [14, 15]. However, there are some contradictions regarding the beneficial anti-tumour effects of TTM. At low 3-μM concentration, it had no significant effect on cell viability but synergised with 10-μM cisplatin against breast cancer cells [14]. In colorectal cancer cells, 10-μM TTM increased expression of the hCTR1 protein in DLD-1 and SW620 cells but only potentiated 100-μM OxPt cytotoxicity in DLD-1 but not in SW620 cells [16]. In prostate cancer, another Cu chelator, clioquinol selectively targeted and rapidly destroyed tumour prostate lines without harming primary prostate epithelial cells but this Cu-dependent toxicity of clioquinol was abrogated by TTM [17]. In other studies, TTM above 2.5 μM inhibited the growth of some androgen-receptor prostate cancer cells [18]. In the same study, the most disappointing finding was that TTM treatment also inhibited the growth of non-neoplastic prostate epithelial RWPE-1 cells, concluding that TTM chelation by itself was not a viable therapeutic option for prostate cancer [18]. However, the same authors found that androgens enhanced Cu uptake and proliferation by prostate cancer cells, but both of these changes were more effectively suppressed by disulfiram (DSF), another FDA-approved Cu ionophore, quoted as most effective when co-administered with Cu [18]. Similar benefits of DSF against prostate cancer were reported by others [19, 20]. However, TTM almost completely blocked DSF-Cu-induced cell death in SUM149 and rSUM149 inflammatory breast cancer cells, highlighting the importance of Cu binding for enhancement of DSF’s cytotoxic effects [20]. Although both DSF and TTM are Cu chelators, TTM decreases intracellular Cu trafficking [22], unlike DSF which can reach other Cu-dependent intracellular proteins [19, 22]. These apparently contradictory data between TTM and DSF imply that antitumour activity is not simple Cu chelation by TTM [13–16] but rather a gain of function seen after DSF is taken up and subsequently is free to redistribute itself by cancer cells, to increase reactive oxygen species production, under the reductive intracellular environment [18–21, 23]. Hence, this report investigated the individual or combined toxicity of TTM and DSF, in combination with some other Cu chelators or with UO126, a specific MEK inhibitor [24, 25] in some human melanomas. Aiming to avoid collateral Cu toxicity, our earlier study aimed to augment Cu chelator without exogenous Cu supplementation [26] using wt p53 C8161 melanoma cells lacking the ^V600E^ BRAF mutation compared to wt p53 A375 human melanoma harbouring this typical BRAF oncogenic mutation [26]. Since then, *genetic analysis showed an atypical* ^G464E^ mutation in the BRAF P loop region, accompanied by an enhancing ^G12D^ KRAS common oncogenic mutation [27] which adds to higher c-MYC expression in C8161 melanoma compared to A375 cells [28]. We also investigated whether other Cu chelators or MEK inhibitors behaved like TTM or DSF in wt p53 melanomas differing in KRAS/c-MYC or BRAF status. Since both KRAS and BRAF mutations drive tumour cell proliferation by Cu-dependent MEK1/2 kinase activation through different responses in melanomas [4] or colorectal carcinomas (CRC) [5], we also studied the response to TTM and/or DSF in mutant p53 CRC with mutant KRAS [29] and high c-MYC [30] compared to CRC cells with a BRAF-mutant [29] low c-MYC [30] status and another mutant p53 CRC harbouring wt BRAF and wt KRAS [29].

## Materials and methods

### Cells

- wt p33 C8161 melanoma has a G464E mutation in the BRAF P loop region, accompanied by an enhancing KRAS G12D mutation [27]. c-MYC expression was found to be six-fold greater in C8161 cells than in A375 cells [28].
- wt p53 A375 melanoma [CRL-1619] with a homozygous BRAF (V600E) mutation was obtained from the American Type Culture Collection [28].
- SW620 colorectal cancer cells harbour two p53 mutations (pR273H; P309S), a KRAS (pG12V) homozygous mutation [29] and have a 6-fold high c-MYC amplification relative to placental DNA [30].
- HT-29 colorectal cancer cells harbour a homozygous p53 mutation (pR273H), a heterozygous BRAF (V600E) mutation [29] and only have a two-fold high c-MYC amplification relative to placental DNA [30].
- Caco-2 colorectal cancer cells harbour a p53 mutation (E204X) and are wild-type for KRAS, BRAF, PIK3CA and PTEN [29]. These cells undergo enterocytic differentiation, decreasing their c-MYC expression in response to butyrate [31]

These cell lines were kept in complete Dulbecco’s containing medium supplemented with 10% foetal calf serum. Although this medium practically does not have Cu supplementation, when supplemented with 10% serum, it provides sufficient copper for cell growth and survival, approx. 50–100 ng/mL since serum albumin is a physiological Cu transporter [http://www.sigmaaldrich.com/life-science/cell-culture/learning-center/mediaexpert/copper.htm]. Whenever indicated, cultures were seeded overnight at 5 × 103 cells per 96 well plates in octuplicates and treatments were added 20 hours after, for a further 24 hours.

### Relative cell viability/metabolic activity

This was estimated with Alamar Blue (resazurin) by measuring intracellular redox mitochondrial activity by quantitating the cell-catalysed conversion of non-fluorescent resazurin to fluorescent resorufin [26]. Alamar Blue was added to a 10% final concentration to each one of 96 well plates after the appropriate treatment. This assay is valuable as an endpoint of proliferation or relative viability/metabolic activity. For these experiments, cells (5,000) were allowed to adhere overnight in 96 well tissue culture plates. After the corresponding treatments, Alamar Blue (BioSource, Camarillo, CA, USA) was added without removing medium containing dead cells and fluorescence measured 4 hours later in a Fluoroskan Ascent microplate reader with an excitation of 544 nm and an emission of 590 nm.

### Quantitation of CRC survival by infrared fluorescence of crystal violet stained cells

Fixed cells surviving after the indicated treatments were washed, then fixed in 70% ethanol. Subsequently, the same cells were stained with crystal violet [26] and the relative ratio of surviving cells was quantitated by crystal violet infrared detection with an Odyssey CL-x infrared imaging system, using *ImageStudio Ver 5.0.21* quantitation software.

### Statistical analysis

All experiments were performed in octuplicates (*n* = 8), *t*-tests were used in Alamar Blue quantitation assays, in which the criteria for statistical significance was taken as *p* s r st, whenever indicated by *. Analysis of Variance (ANOVA) tests with Tukkey *posteriori* analysis were used for infrared quantitation of crystal violet stained CRC cells, in which the criteria for statistical significance were also taken as *p* s o ta, whenever indicated by *.

## Results

### Toxicity induced by neocuproine ± DSF is similarly reversed by TTM in both C8161 and A375 cells

**Figure 1.**
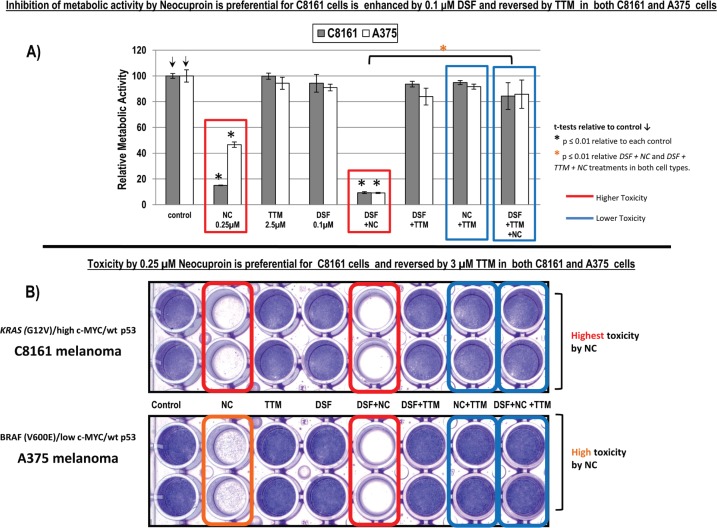
(a): Changes in metabolic activity/cell viability were estimated in sub-confluent cells seeded overnight followed by exposure to the treatments indicated for 72 hours in 96 well plates (*n* = 8), using the Alamar Blue resazurin/resorufin fluorometric assay described under methods. Results shown are representative of three different assays. (b): Differences in cell survival were assayed after the indicated treatments for 72 hours by fixing cells with 70% ethanol and staining with crystal violet, as described under methods.

We used neocuproine (NC), another membrane permeable Cu (I) chelator, also known as 2, 9-dimethy l−1, 10-phenanthroline [32] to ask whether: a) it also competed with DSF and b) its toxicity was also antagonised by 3-μM TTM. These studies revealed that: a) 0.25-μM NC was similarly toxic to both melanoma types, b) its activity was further enhanced by 0.1-μM DSF and c) that its activity was blocked by 3-μM TTM, even when NC was added together with DSF (Figure 1a and b).

### Toxicity by co-treatment with sublethal DSF and the copper (II) chelator 1, 10-phenanthroline is reversed preferentially by 2.5-μM TTM in A375 cells compared to C8161 cells

**Figure 2.**
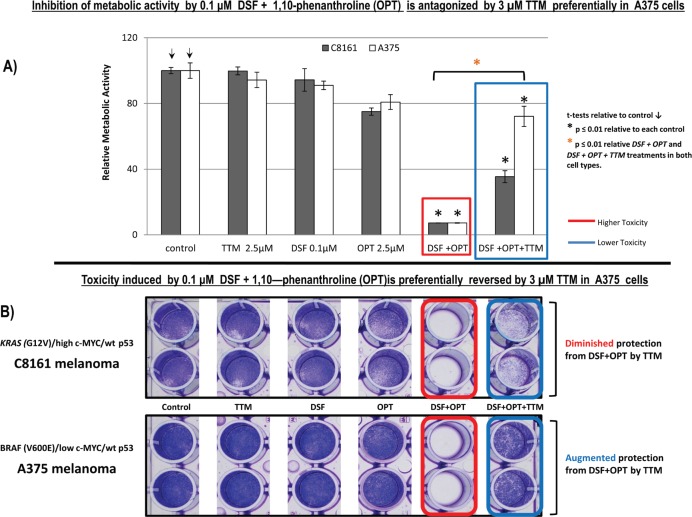
(a): Sub-confluent cells seeded overnight in octuplicates were exposed to the treatments indicated for 72 hours in 96 well plates (*n* = 8). Changes in metabolic activity/cell viability were then measured fluorometrically with Alamar Blue. Results shown are representative of three different assays. (b): Differences in cell survival were assayed after the indicated treatments for 72 hours by fixing cells with 70% ethanol and staining with crystal violet, as described under methods.

1, 10-orthophenanthroline (1, 10-OPT), a cell-permeable chelator of Cu2+ [33, 34], was also tested for its anti-melanoma activity at 2.5 μM, either by itself or in conjunction with 0.1 DSF and/or with 2.5-μM TTM. As expected, no significant toxicity was observed in metabolic activity assays or in survival crystal violet assays when C8161 or A375 cells were exposed to 0.1-μM DSF, unless it was supplemented with 2.5-μM 1, 10-OPT. However, the addition of 2.5-μM TTM once again reversed the toxicity induced by co-treatment with 0.1-μM DSF and 2.5 μM 1, 10-OPT. However, TTM attenuation of the toxicity of the other two Cu chelators was greater in ^(V600E) *mut BRAF*^ A375 cells compared to ^G12V-mut *KRAS / high c-MYC*^ C8161 cells (Figure 2a and b).

### Toxicity by co-treatment with sublethal DSF and MEK inhibitor, UO126 is reversed by TTM in both C8161 and A375 cells

**Figure 3.**
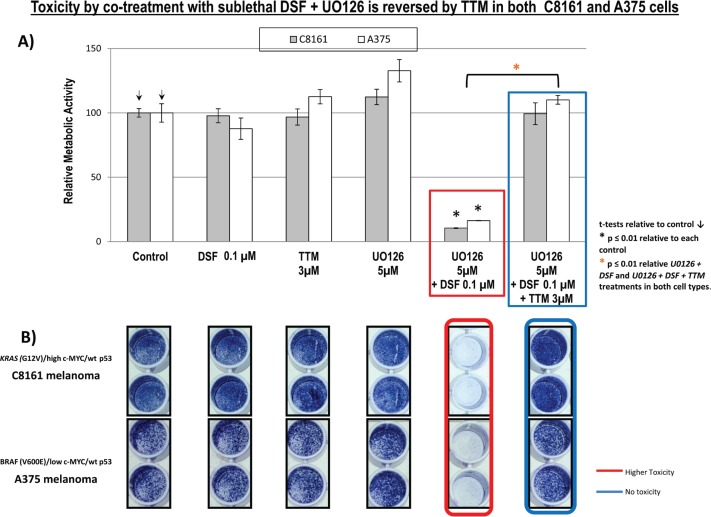
(a): Changes in metabolic activity/cell viability were estimated in sub-confluent cells seeded overnight followed by exposure to the treatments indicated for 72 hours in 96 well plates (*n* = 8), using the Alamar Blue resazurin/resorufin fluorometric assay described under Methods. Results shown are representative of three different assays. (b): Differences in cell survival were assayed after the indicated treatments for 72 hours by fixing cells with 70% ethanol and staining with crystal violet, as described under methods.

Since Cu chelation decreases the Cu dependence of MEK1/2 activation for KRAS [1] or BRAF [2, 3] optimal oncogenic signalling, and U0126 selectively binds and inhibits MEK-1/2 [24] but also protects from oxidative stress [25], we hypothesised that sub-toxic Cu chelation together with UO126 would diminish non-specific toxicity preserving anti-tumour activity against melanoma cells with KRAS or BRAF mutations. Using 100-nM DSF or 5-μM UO126 as single agents did not suppress metabolic activity or survival of C8161 or A375 cells. However, these cells greatly diminished metabolic activity adding together the indicated sub-lethal concentrations of these two agents, in a reaction reversed by TTM (Figure 3a and b).

### Toxicity induced by co-treatment with sublethal DSF ± OxPt is attenuated by TTM in Caco-2 cells but not in SW-620 cells

**Figure 4.**
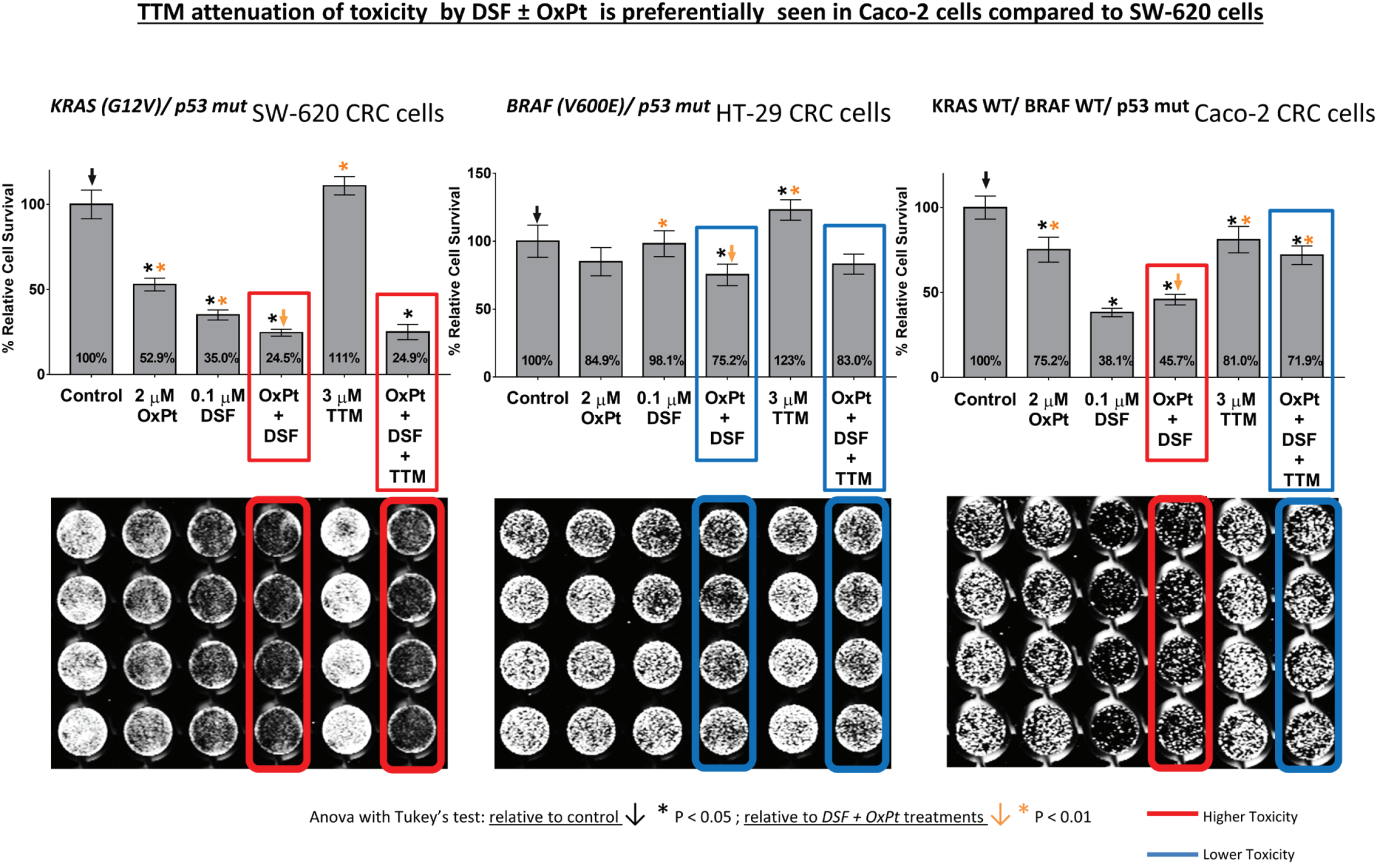
Differences in cell survival were estimated in colorectal cancer cells seeded to subconfluency overnight followed by exposure to the indicated treatments for 72 hours in 96 well plates (*n* = 8), followed by fixing cells with 70% ethanol, staining with crystal violet and infrared quantitation as described under methods. Results shown are representative of three different assays. Note the significant toxicity to DSF ± OxPt, which was not counteracted by TTM in ^KRAS (G12V)/ p53 mut^ SW-620 cells (left). Attenuated response to DSF ± OxPt with or without TTM in ^BRAF (V600E)/ p53 mut^ HT-29 cells (centre) and growth inhibition by DSF ± OxPt, partly antagonised by TTM in ^KRAS WT/ BRAF WT/ p53 mut^ Caco-2 cells (right).

Since the response to cetuximab in CRC is impaired by KRAS/BRAF mutations [4–6], these do not affect their response to OxPt [7, 8] since the latter is incorporated through the hCTR1 transporter activated by Cu chelation. Hence, three different CRC were assayed for their response to DSF ± OxPt in the presence or absence of 3-μM TTM. There was no toxicity against the three CRC tumour cells tested when using TTM as a single treatment. Survival in *^KRAS (G12V)/p53 mut^* SW-620 cells with high c-MYC amplification by single OxPt approximated 52.9%, with DSF treatment permitting a 35% survival, which was further decreased when DSF + OxPT (24.5%) and was not counteracted by TTM (Figure 4, left). No comparably significant toxicity in response to DSF ± OxPt with or without TTM was evident in ^BRAF (V600E)/ p53 mut^ HT-29 cells with a lower c-MYC amplification (Figure 4, centre). In contrast, significant growth inhibition by DSF alone or when combined with OxPt (45.7 %) was partly antagonised by TTM (71.9 %) resembling the greater survival seen with OxPt treatment in *^KRAS WT/ BRAF WT/ p53 mut^* Caco-2 cells (Figure 4, right).

## Discussion

This study examined whether anti-cancer response was enhanced or interfered with by co-treatment between two structurally different Cu chelators currently used in clinical trials like a) TTM [A Phase II Study of *Tetrathiomolybdate* in Patients With Breast Cancer at Moderate to High Risk of Recurrence. ClinicalTrials.gov Identifier NCT00195091; study completion date: June 2020] and b) DSF [Copper Chloride, *Disulfiram*, and Copper Gluconate in Treating Patients with Metastatic Castration-Resistant Prostate Cancer. Clinicaltrials.gov Identifier NCT02963051 study completion date: August 2020] and Phase II Trial of *Disulfiram* With Copper in Metastatic Breast Cancer ClinicalTrials.gov Identifier NCT03323346; study completion date September 2020]. Since KRAS mutation and c-Myc amplification cooperate with KRAS in tumourigenesis [35–37], this report used cell lines differing in BRAF, KRAS and C-MYC status to gain insight into their modulation of response to Cu chelators. We found that 2.5-μM TTM or low 0.1-μM DSF did not suppress growth and metabolic activity in two wt p53 human melanoma cells harbouring KRAS mutation and high c-MYC (C8161) or BRAF mutation and low c-MYC (A375) (Figure 1). In contrast, 0.25-μM NC—another Cu chelator—preferentially inhibited metabolic activity to approximately 20% of its control in C8161 cells with KRAS mutation and high c-MYC expression compared to a decrease to about 40% of its control in A375 cells with BRAF mutation and low c-MYC (Figure 1a). More importantly, the synergism between two Cu chelators like NC and 0.1-μM DSF cells was notably reversed by TTM, another Cu chelator, to a similar extent in both C8161 and A375 melanoma cells (Figure 1).

**Figure 5.**
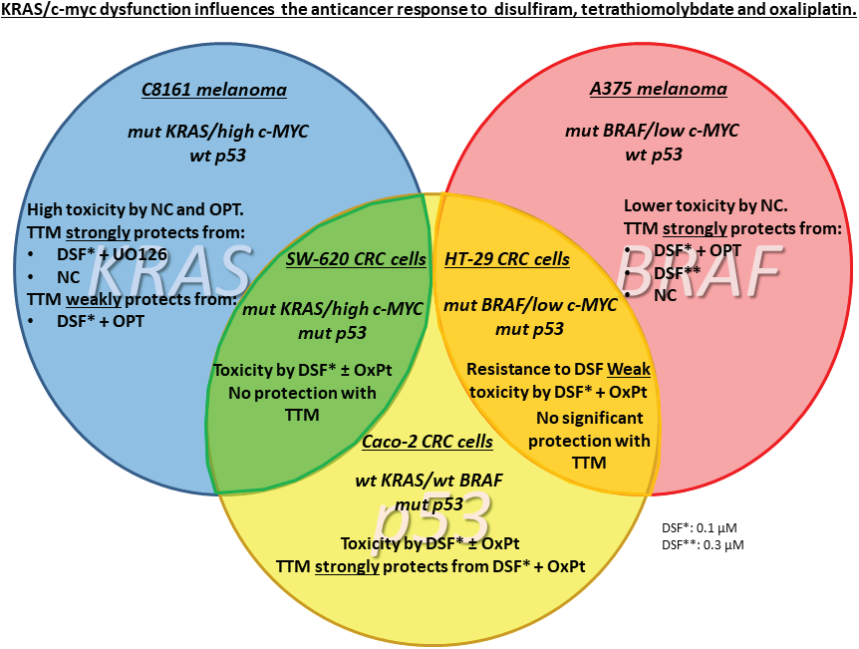
Summary. KRAS/c-myc dysfunction influences the anticancer response to DSF, TTM and OxPt.

**Figure S1.**
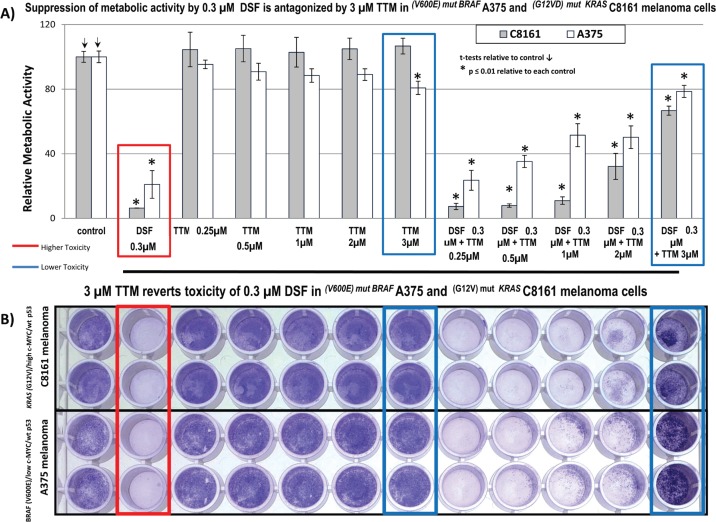
(a): Suppression of metabolic activity by 0.3-μM DSF is antagonised by 3-μM TTM in *^(V600E) mut BRAF^* A375 and *^(G12VD) mut KRAS^* C8161 melanoma cells. (b). 3-μM TTM reverts toxicity of 0.3-μM DSF in *^(V600E) mut BRAF^* A375 and ^(G12V-)mut ^*^KRAS^* C8161 melanoma cells.

However, when using another Cu chelator like 0.25-μM OPT, it preferentially killed C8161 cells compared to A375 cells. However, in C8161 cells with concomitant KRAS/c-MYC dysregulation, TTM reversion of melanoma toxicity by DSF + OPT (Figure 2) was diminished. We also show for the first time that the synergism between sub-lethal levels of DSF and the highly specific MEK inhibitor UO126 [24] against melanoma cells, irrespective of BRAF or KRAS/c-MYC status, This synergism may be partly explained by DSF chelating activity sequestering Cu [21] required as a MEK1/2 co-activator in KRAS-[1] or BRAF-mediated oncogenic signalling [2, 3] and MEK inhibitors like UO126 [24] directly binding to MEK1/2. Such synergism was also antagonised by TTM (Figure 3). Moreover, TTM not only inhibited the ability of 0.1-μM DSF to synergise with other Cu chelators (Figures 1–3) but also when DSF used at the toxic 0.3-μM concentration against C8161 and A375 cells (Figure S1). The different response between Cu chelators like DSF and TTM to MEK inhibitors or OxPt may be partly linked to the latter decreasing intracellular Cu trafficking [22], whereas DSF promotes Cu intracellular redistribution and greater bioavailability [19]. Although exogenous Cu supplementation has been widely used by others to augment the efficacy of TTM [3, 12–15] and DSF [18, 20, 21], we reported that DSF was much more effective than TTM as an anticancer agent even without Cu supplementation to avoid collateral Cu toxicity [26]. Another study investigating if elevated copper enhances the efficacy of the anticancer drug, imatinib (ITB), also showed that DSF was more effective than high Cu (II) as an adjuvant to ITB [37], confirming our belief that restrained manipulation of copper level in tumour may lead to a more selectively targeted killing of tumour cells and diminished collateral toxicity. DSF also showed greater efficacy against SW-620 and Caco-2 CRC cells, compared to the relatively greater resistance to DSF ± OxPt in *^BRAF (V600E)/ p53 mut^* HT-29 CRC cells. It was also noteworthy that TTM attenuated toxicity by DSF ± OxPt preferentially in ^KRAS WT/ BRAF WT/ p53 mut^ Caco-2 cells compared to ^*KRAS (G12V)/ p53 mut/ high* c-MYC^ SW-620 cells (Figure 4). Although this study is still at an early stage, our results suggest that mutant KRAS/high c-MYC amplification [38] may change the anti-tumour response to some specific Cu chelators in wt p53 C8161 and A375 melanoma, which are compatible with others who found that c-MYC deregulation without elevated expression cooperates with KRAS^G12D^ mutation to accelerate tumourigenesis [35, 36, 38]. *However, the cooperation between mutant KRAS and extent of c-MYC amplification may be different in the CRC cells harbouring a mutated tumour suppressor p53 gene, used in these studies* (Figure 5). Taken together, our findings are the first to show that Cu sequestration may be necessary but not sufficient for anti-cancer activity, given that TTM which binds but does not release intracellular Cu [19, 22] did not significantly inhibit any of the tumour cells tested but rather suppressed the inhibition caused by other Cu chelators.

## Conclusion

In summary, our studies imply that DSF may be the most clinically promising anti-cancer Cu chelator. DSF re-purposing from its long-term clinical use against alcoholism to an anti-cancer drug is based on its ability to act as an ALDH1A1/ALDH2 inhibitor [39], a property not shared by other Cu chelators like TTM. ALDH enzymes are implicated in the breakdown of acetaldehyde to acetate, an obligatory step in alcohol metabolism. DSF inhibition of ALDH1A1/ALDH2 activity also prevents removal and increases cellular acetaldehyde accumulation, and selective DNA damage, both in proliferating and slower replicating cancer stem cells, which frequently have genomic instability and high oxidative stress [39, 40] in contrast to their normal cell counterparts.

## Author contributions statement

Ali Calderon-Aparicio carried out the melanoma experiments, cooperated with Alejandro Cornejo in the CRC experiments and helped in the Discussion. Alejandro Cornejo carried out the statistical analyses, designed the Graphic Abstract and helped to improve the Discussion. Andrea Orue participated in the Discussion and final revision of this manuscript. Manuel Rieber designed, supervised, provided funding and wrote the final version of this paper.

## Financial disclosure

This research was funded by Fonacit-Misión Ciencia sub-proyecto Sistema Publico Nacional de Salud 4-Cancer grant to Manuel Rieber at Instituto Venezolano de Investigaciones Cientificas. The funders had no role in the study design, data collection and analysis, decision to publish or preparation of the manuscript.
